# The influence of tactical positioning on performance in world-championship freestyle swimming

**DOI:** 10.3389/fspor.2025.1600554

**Published:** 2025-06-09

**Authors:** Craig Staunton, Jesús J. Ruiz-Navarro, Peter Edholm, Dennis-Peter Born

**Affiliations:** ^1^ Department of Environmental and Bioscience, School of Business, Innovation and Sustainability, Halmstad University, Halmstad, Sweden; ^2^ Aquatics Laboratoty, Department of Physical Education and Sports, Faculty of Sport Sciences, University of Granada, Granada, Spain; ^3^ Swiss Swimming Federation, Swiss Development Hub for Strength and Conditioning in Swimming, Worblaufen, Switzerland; ^4^ Department for Elite Sport, Swiss Federal Institute of Sport Magglingen, Magglingen, Switzerland

**Keywords:** pacing, performance analysis, rank order, race strategy, positioning

## Abstract

**Purpose:**

This study examined tactical positioning and pacing in short-distance (100 m), middle-distance (200 m, 400 m) and long-distance (800 m, 1,500 m) freestyle events, focusing on the influence of race distance, competition round (HEAT vs. FINAL), and sex.

**Methods:**

Race data from multiple World Championships (2013–2023) were analyzed. Spearman's rank correlations (ρ) were calculated to examine rank stability across race distances, competition rounds, and sex. Additionally, lane distributions of Top3 finishers were analyzed to assess the impact of lane position on race outcomes.

**Results:**

Rank correlations increased progressively from the first to the final lap across all race distances (*p* < 0.05). Long-distance events exhibited earlier rank stabilization, with correlations reaching *ρ* ≥ 0.90 by 50% race completion, whereas middle-distance events showed greater positional variability throughout. Rank correlations were lower in FINALS than in HEATS (*p* < 0.05), indicating greater positional shifts in high-stakes races. No significant sex-based differences were observed (*p* > 0.05). Central lanes (4 and 5) were associated with the highest Top3 finish rates, while outer lanes (0, 1, 8, and 9) had the lowest, particularly in long-distance events.

**Conclusion:**

Finals foster more dynamic race strategies, with increased positional changes. Rank stability was achieved at a relatively earlier proportion of the race in long-distance events compared to middle-distance events. By the final lap, rank stability converged across all distances, suggesting that race order was largely set before the last lap, emphasizing early tactical positioning over late-race surges. These findings offer insights into optimizing race tactics and pacing in elite swimming.

## Introduction

Competitive swimming events at the national and international level require athletes to advance through multiple rounds, including heats, semifinals, and finals, depending on the competition format (1). Swimmers must use strategic race tactics, including tactical positioning and pacing, to advance and secure a favourable lane assignment for subsequent rounds, while avoiding unnecessary fatigue. Tactical positioning refers to how swimmers strategically position themselves relative to their competitors during the race to optimize drafting benefits, control pacing, and execute effective finishing strategies. Prior research has demonstrated that swimmers in the middle lanes (typically lanes 4 and 5) tend to have a competitive advantage, partly due to reduced hydrodynamic disturbances and better race visibility (2). However, the influence of tactical positioning on race outcomes, particularly across different race distances and sexes, remains an area of ongoing investigation.

One of the primary hydrodynamic factors affecting competitive swimmers is wave drag, which increases water resistance and can significantly impact race performance (3). This form of resistance occurs as swimmers generate waves while moving through the water, creating additional drag that can hinder forward motion and influence overall speed. In this sense, international swimming regulations mandate the use of wave-breaking lane ropes in official long-course races (50 m pool length) to minimize wave disturbances between the ten swimming lanes (1). However, despite the 2.5 m wide lanes separated by lane ropes with 0.10–0.15 m thick floats (1), swimmers still remain susceptible to the wake effects generated by nearby competitors. While disturbed water may impair propulsion and disrupt stroke mechanics at hand entry and catch positions (4), some swimmers may strategically utilize the wake of a competitor ahead of them to reduce drag and conserve energy (known as drafting) (5, 6). This drafting effect is well-understood and applied in open-water swimming and may also be present in pool-based competitions (5, 6), despite lane separators designed to mitigate these interactions.

Tactical positioning is especially critical in sprint events (50 m and 100 m freestyle), where swimmers typically adopt a positive pacing strategy—starting fast to gain advantage by securing an early favourable position leading position (7). The importance of early-race performance as a key performance indicator has been highlighted in previous research, with positioning at the 15 m mark often aligning with final race standings (8, 9). Given that sprint races generate significant wave interactions, securing a position ahead of competitors early on may be crucial for success. However, tactical positioning and pacing may also play a role in middle- and long-distance freestyle events (200 m, 400 m, 800 m, and 1,500 m), where swimmers employ various approaches such as the fast-start-even strategy or the fast-start-settle-fast-finish strategy (10–15). Previous research has indicated that performance levels differ between heats, semifinals, and finals, with swimmers often adjusting their pacing and effort distribution to optimize advancement through rounds whilst minimising fatigue (16). Additionally, for freestyle swimming events, middle lanes may offer a strategic advantage by providing swimmers with a better overview of their competitors, enabling them to adjust their pacing strategy dynamically based on the race situation. Since lane assignments in finals are based on qualification times from previous rounds, the relationship between swimming lane and final race standing warrants further investigation.

Despite advancements in understanding pacing strategies, research on tactical positioning remains limited, particularly in analysing how competitive performance across different rounds is associated with final performance in elite swimming. Moreover, while studies have examined pacing differences and performance variability between finalists and non-qualified swimmers (17–19), fewer studies have investigated the impact of tactical positioning across different race distances. Some research has explored similar concepts in other sports, such as cross-country skiing (20) and distance running (21, 22), yet this understanding remains limited within swimming. Moreover, most studies have been conducted in male swimmers, which highlights the necessity to investigate sex-specific differences. Such knowledge is essential for athletes and coaches to be able to select the most effective strategy and tactical positioning, ultimately optimizing performance and competitive outcome.

As such, this study was exploratory in nature, aiming to better understand tactical positioning and pacing strategies in world championship freestyle swimming. Specifically, we examined differences between heats and finals, middle- and long-distance events, and potential sex-based variations. Without a predefined hypothesis, our goal was to generate new insights into race tactics and the strategic role of positioning in elite-level performance, ultimately offering practical guidance for swimmers and coaches.

## Methods

### Subjects

Race results and split times of all freestyle swimmers from the 2013, 2015, 2017, 2019, 2022 and 2023 long-course (50 m pool length) World championships were provided by the Swimrankings database (https://www.swimrankings.net, Bern, Switzerland). All races were compliant with competition regulations stipulated by the International Swimming Federation (World Aquatics). The 2024 World Championships were deliberately excluded from the analysis due to the atypical scheduling of the event in an Olympic year, which led to the absence of several top-ranked swimmers prioritizing Olympic preparation. In total, data from 5,103 races were analysed including heats (*n* = 4,241), semi-finals (*n* = 382) and finals (*n* = 480). The study was pre-approved by the institutional review board of the Swiss Federal Institute of Sport Magglingen (Reg.-Nr. 222_LSP_Born_03_2024). The study was conducted in accordance with the code of conduct of the World Medical Association for research involving human subjects (Declaration of Helsinki).

### Data collection and analysis

Race data were analyzed for all freestyle events for both women and men. Swimmers were categorized based on whether they finished in the Top3 of their respective race (Top3) or not (non-Top3), including results from both heats (HEAT) and finals (FIN).

Tactical positioning was conceptualized as the swimmer's relative position (i.e., ranking) in the pool at various stages of the race. Pacing strategy was indirectly assessed by examining how rankings changed across 50 m splits in different race distances and competition rounds (heats vs. finals). This permitted inference of tactical positioning and pacing behaviours and how swimmers adjusted their speed relative to their competitors at various stages of the race.

To explore tactical positioning and pacing strategy, the rank at each 50 m split was analyzed. Rankings were compared between Top3 and non-Top3, across different race distances, and between women and men. As a complementary analysis, the proportion of Top3 rankings from each individual lane was computed for all race distances.

Only race distances of 200 m or greater (i.e., 200 m, 400 m, 800 m & 1,500 m) were included in analyses of tactical positioning and pacing to allow for correlation analyses between intermediate split rank and final rank with a meaningful number of 50 m splits. Although semifinals exist in the 200 m event, they were excluded from analysis because no comparable semifinals are held for the 400 m, 800 m, or 1,500 m events, ensuring consistency across race distances. The 200 m and 400 m distances were considered middle-distance events and 800 m and 1,500 m distances were considered long-distance events. For the analysis of the Top3 distribution across the ten pool lanes, all race distances (including 50 m and 100 m) were considered, as this analysis does not depend on intermediate split times.

### Statistical analysis

Spearman rank correlation coefficients (ρ) were calculated to assess the relationship between intermediate 50 m split ranks (i.e., ordinal variables) with final ranking across different competition rounds (HEAT and FIN). As these data are not continuous, Pearson correlation analyses were not appropriate. To statistically compare the strength of relationships between race distances, sexes, and competition stages (HEAT vs. FIN) and to assess how these relationships evolved over different race segments, Spearman rank correlation coefficients were z-transformed using Fisher's z-transformation to allow parametric statistical comparisons (23). For ANOVA analyses, race data were divided into five segments to examine ranking dynamics over time: first lap, first third, middle third, last third, and final lap. The 200 m freestyle was treated as a special case, where the first third and last third were excluded, as they overlapped with the first and final lap. To account for the unequal number of TIMING levels across distances, analyses were conducted separately for the 200 m and the longer events. As such, two separate mixed-design ANOVAs were conducted on the z-transformed rank correlations. ROUND (HEAT vs. FIN), DISTANCE (200 m, 400 m, 800 m, 1,500 m), and TIMING (race segments) were treated as within-subject factors, while SEX (women vs. men) was treated as a between-subject factor. Given differences in race structure between the 200 m and longer distances (≥400 m), we defined TIMING differently for these two groups: For distances of 400 m and longer, TIMING was divided into five segments: first lap, first third, middle third, last third, and final lap. A two × three × two × five repeated-measures ANOVA (SEX × DISTANCE × ROUND × TIMING) was conducted. For the 200 m event, TIMING was defined using three segments: first lap, middle third, and final lap, since the first third and last third were redundant with the first and final lap. A separate two × two × three repeated-measures ANOVA (SEX × ROUND × TIMING) was conducted for this event.

Significant interactions were explored using *post hoc* pairwise comparisons with Bonferroni correction. Effect sizes were reported as partial eta squared (η^2^_p_). Normality was primarily assessed using visual inspection of Q-Q plots and histograms, supplemented by the Shapiro–Wilk test. Given the large sample sizes, visual methods were prioritized in guiding decisions on the appropriate statistical tests. Mauchly's test of sphericity was consulted, with Greenhouse-Geisser corrections applied where the assumption of sphericity was violated. All statistical analyses were performed using SPSS 27.0 (IBM, Chicago, IL, USA), with significance set at *p* < 0.05. Spearman rank correlation coefficients (ρ) are presented as median ± interquartile range (IQR) since ordinal data are more appropriately summarized using median and IQR. Z-transformed rank correlation coefficients are presented as mean ± standard deviation.

## Results

**Figure 1:**
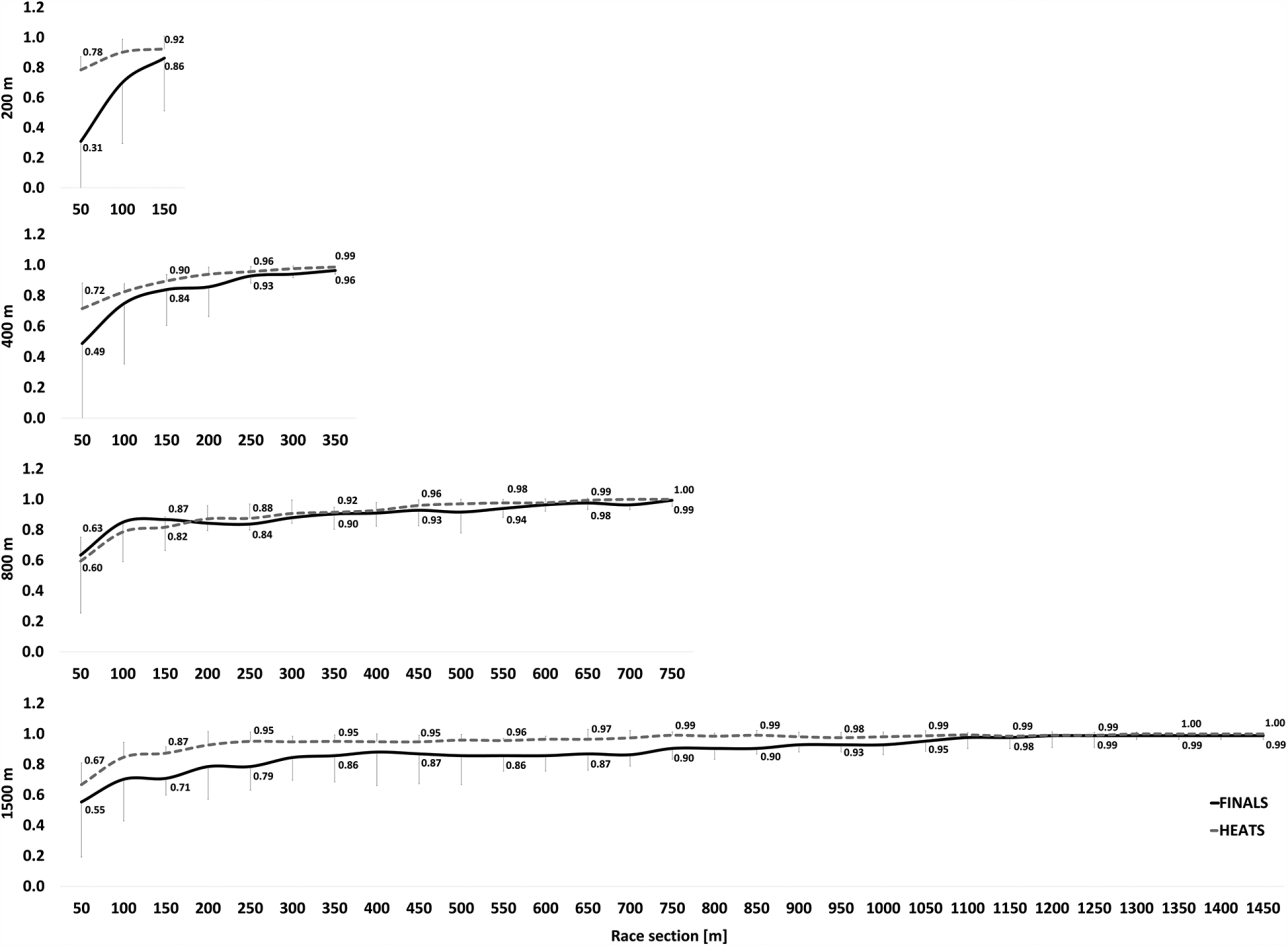
Median of Spearman's correlation coefficient ± interquartile range between the positioning at the various 50 m splits and the final rank for *female* freestyle swimmers. For the sake of readability, correlations coefficients are only indicated for every second split time.

**Figure 2:**
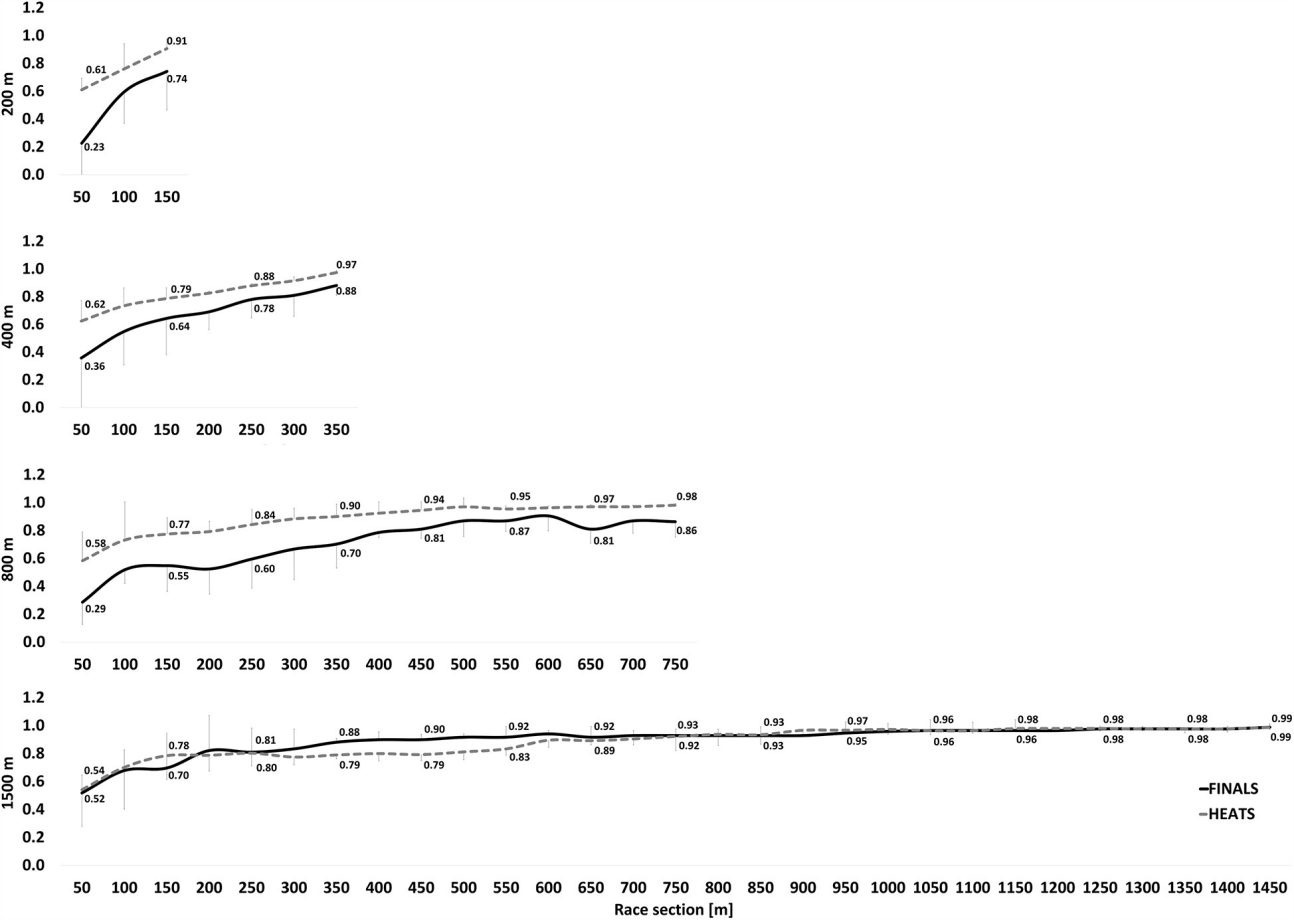
Median of Spearman's correlation coefficient ± interquartile range between the positioning at the various 50 m splits and the final rank for male freestyle swimmers. For the sake of readability, correlations coefficients are only indicated for every second split time.

The rank correlation coefficients for each race distance are displayed in Figure 1 for women and Figure 2 for men. Table 1 shows the z-transformed rank coefficient data with statistical analyses.

**Table 1:** Mean of the fisher's *z*-score transformed rank correlation coefficients between positioning in the particular race sections and the final race ranking (± standard deviation).

	First lap	First third	Middle third	Last third	Last lap
Women					
FIN					
200 m	0.40 ± 0.63	–	0.72 ± 0.48	–	1.23 ± 0.69
400 m	0.71 ± 0.66	0.88 ± 0.63	1.46 ± 0.53	1.98 ± 0.44	2.08 ± 0.45
800 m	0.78 ± 0.57	1.09 ± 0.54	1.57 ± 0.48	2.05 ± 0.38	2.29 ± 0.54
1,500 m	0.87 ± 0.56	1.17 ± 0.51**	1.48 ± 0.33**	2.13 ± 0.49***	2.37 ± 0.33
HEAT					
200 m	1.13 ± 0.24*	–	1.39 ± 0.28*	–	1.71 ± 0.3*
400 m	0.95 ± 0.33	1.16 ± 0.33*	1.78 ± 0.29*	2.28 ± 0.22*	2.40 ± 0.16*
800 m	0.77 ± 0.32	1.17 ± 0.25*	1.87 ± 0.24*	2.29 ± 0.14*	2.45 ± 0.14*
1,500 m	0.74 ± 0.35	1.62 ± 0.26*^,^**	2.21 ± 0.27*^,^**	2.46 ± 0.15*^,^***	2.58 ± 0.09*
Men					
FIN					
200 m	0.24 ± 0.53	–	0.47 ± 0.48	–	0.88 ± 0.39
400 m	0.36 ± 0.45	0.46 ± 0.42	1.02 ± 0.51	1.57 ± 0.56	1.71 ± 0.59
800 m	0.41 ± 0.25	0.65 ± 0.23	1.15 ± 0.22	1.43 ± 0.32	1.70 ± 0.61
1,500 m	0.63 ± 0.43	1.17 ± 0.21**	1.68 ± 0.33**	2.11 ± 0.35***	2.37 ± 0.33
HEAT					
200 m	0.71 ± 0.20*	–	0.93 ± 0.18*	–	1.41 ± 0.24*
400 m	0.63 ± 0.12	0.77 ± 0.19*	1.22 ± 0.27*	1.88 ± 0.25*	2.10 ± 0.20*
800 m	0.70 ± 0.29	1.07 ± 0.24*	1.59 ± 0.27*	2.00 ± 0.17*	2.25 ± 0.26*
1,500 m	0.56 ± 0.32	1.06 ± 0.31*^,^**	1.76 ± 0.27*^,^**	2.15 ± 0.25*^,^***	2.36 ± 0.19*

FIN, finals; HEAT, heats.

* Different to FIN (*p* ≤ 0.015).

** Different to 800 m and 400 m events (*p* ≤ 0.020).

*** Different to 800 m only (*p* = 0.025).

For races of 400 m and longer, there were no significant three-way or four-way interactions. However, a significant TIMING × ROUND interaction was observed [F_(4,40)_ = 4.520, *p* = 0.024, η^2^_p_ = 0.311]. Specifically, at the first timing point, there were no significant differences between HEATS and FINALS (*p* = 0.318). However, at all subsequent time points, z-transformed rank correlations were significantly greater in HEATS compared to FINALS (*p* ≤ 0.015). Furthermore, in both HEATS and FINALS, *z*-scores significantly increased across the race from the first to the final timing point (*p* < 0.001).

A significant DISTANCE × TIMING interaction was also found [F_(8,80)_ = 2.993, *p* = 0.047, η^2^_p_ = 0.230], indicating differences in rank correlations across distances at various stages of the race. There were no significant differences between distances at the first timing point (*p* > 0.999). However, at the second and third timing points, the 1,500 m race displayed significantly greater *z*-scores than both the 800 m (*p* ≤ 0.024) and 400 m races (*p* ≤ 0.020). At the fourth timing point, *z*-scores for the 1,500 m were still significantly higher than for the 800 m (*p* = 0.025), but no longer different from the 400 m (*p* = 0.164). By the final timing point, there were no significant differences between distances (*p* ≥ 0.072). Across all race distances, *z*-scores significantly increased from the first to the last timing point, reflecting a general trend of increasing rank consistency throughout the race (*p* < 0.001). There were no significant interactions involving SEX, suggesting that pacing consistency and race dynamics evolved similarly in male and female swimmers.

For the 200 m race, no significant three-way or two-way interactions were detected. However, there were significant main effects for ROUND [F_(1,10)_ = 14.839, *p* = 0.003, η^2^_p_ = 0.597] and TIMING [F_(2,20)_ = 58.778, *p* < 0.001, η^2^_p_ = 0.855], with *z*-scores significantly increasing across the three available timing points. Additionally, HEAT *z*-scores were consistently greater than FINAL *z*-scores, suggesting a stronger rank consistency in heats compared to the finals (*p* = 0.003).

Table 2 displays the proportion of Top3 finishes from each of the 10 pool lanes. The analyses of the proportion of Top3 finishes are descriptive and exploratory, providing insight into the distribution of finishes across lanes for each race distance. These data are not subjected to statistical testing, as the goal is to offer valuable practical information for coaches and athletes. As expected, lanes 4 and 5, assigned to the fastest qualifiers, had the highest proportion of Top3 finishes across all distances. Lane 3 frequently outperformed lane 6, despite both being assigned to similar seed times, suggesting that lane 3 might offer some advantage. Lanes 6 and 7 consistently outperformed lanes 0, 1, 8, and 9, especially in longer races (800 m, 1,500 m). Outside lanes (0, 1, 8, 9) had the lowest Top3 finish rates. Lane data suggest that lane placement effects were slightly stronger in women's events, particularly in longer races, where the vast majority of Top3 finishers came from lanes 4 and 5.

**Table 2:** Average proportion of the winners across the ten pool lanes.

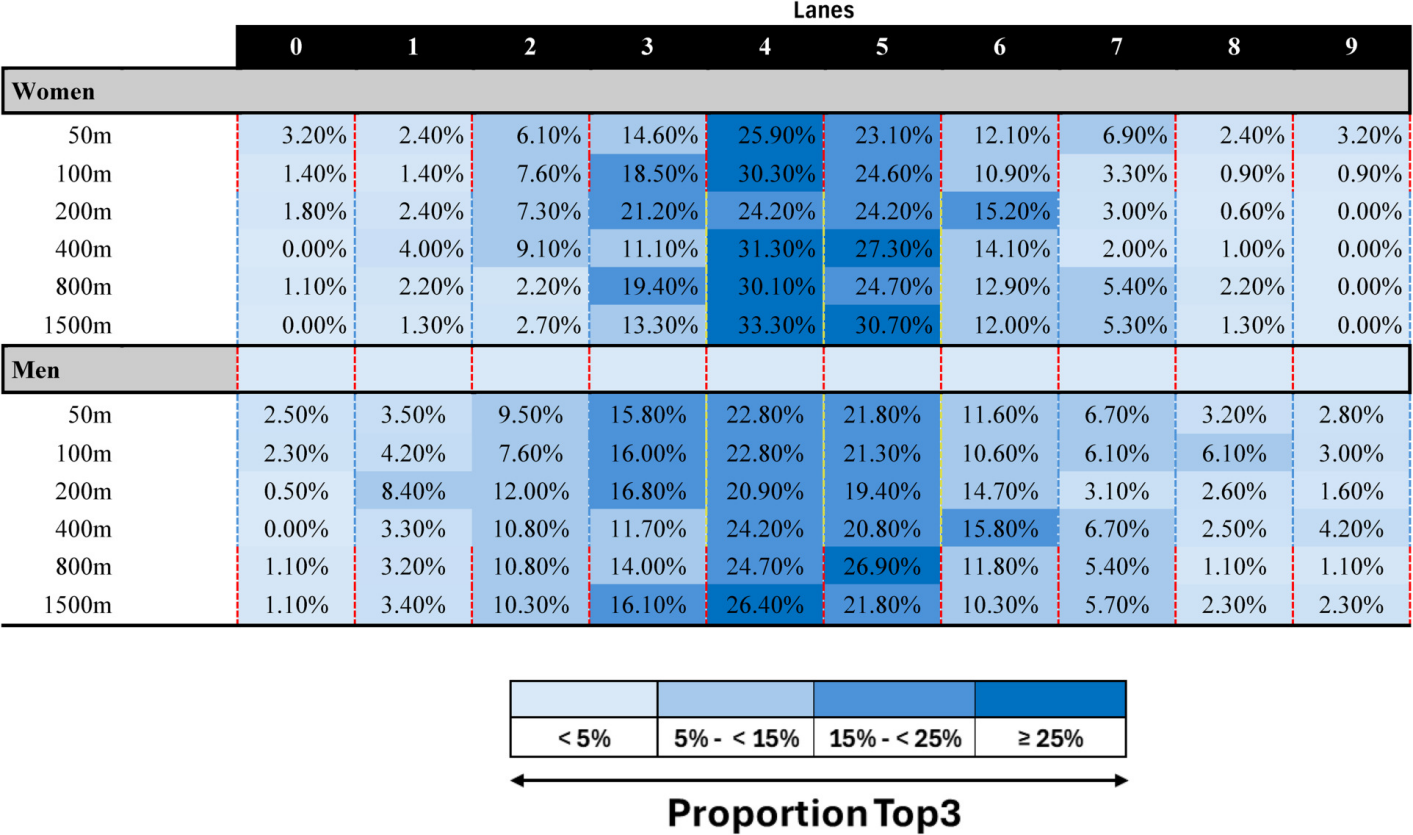

Proportions were averaged from heats and finals.

**Figure 3:**
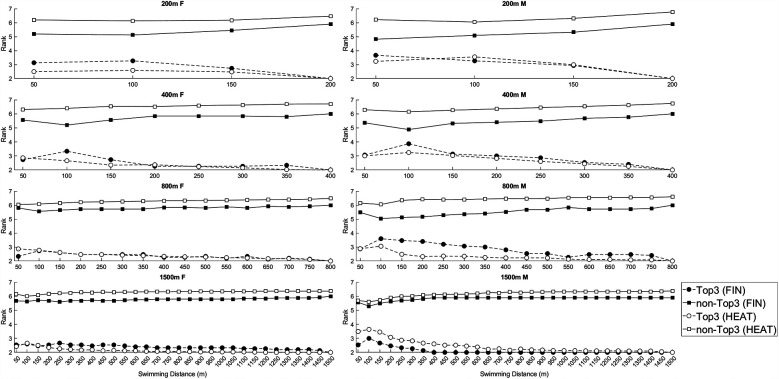
Mean rank at each 50 m split for the top 3 and non-top 3 for all race distances for both women and men. FIN, finals; HEAT, heats; Top3, swimmers who finished in the top 3 positions within their race; non-Top3, swimmers who did not finish in the top 3 positions within their race.

Figure 3 illustrates the rank progression of the Top3 finishers compared to the rest of the field, showing how rankings evolved between HEAT and FIN rounds across all race distances for women and men. Mean and standard deviation rank values at each 50 m split are presented separately for the Top3 and non-Top3 finishers. Across all race distances, Top3 finishers held better rankings throughout the race compared to non-Top3 swimmers. In longer events (800 m and 1,500 m), Top3 finishers maintained relatively stable rankings with minimal variation. In contrast, shorter events (200 m and 400 m) showed greater rank fluctuations, particularly among non-Top3 swimmers. Comparisons between HEAT and FIN rounds showed that Top3 finishers in the finals consistently held better rankings than in the heats. Rank variability was lower in the finals, particularly among non-Top3 swimmers. Women and men showed similar ranking trends.

## Discussion

This study examined tactical positioning and pacing in elite World Championship freestyle swimming races (200 m and longer), with a focus on how race distance, competition round (HEAT vs. FINAL), and sex influenced the consistency of rankings throughout the race. Additionally, we analysed lane distributions of Top3 finishers and the rank progression of Top3 swimmers vs. the other competitors. The key findings were: (1) Rank correlations were consistently lower in finals compared to heats, indicating greater rank fluctuations in the final races. (2) Across all race distances, rank consistency increased from the start to the finish of the race. (3) Long-distance events (800 m and 1,500 m) displayed greater rank stability throughout the race, with stability occurring earlier, compared to middle-distance events (200 m and 400 m), where rank fluctuations were more pronounced. (4) Lane placement influenced Top3 finishes, with central lanes (4 and 5) showing the highest success rates, while outside lanes (0, 1, 8, and 9) had the lowest Top3 finish rates, particularly in long-distance events. (5) The overall trends in rank stability and lane effects were similar between women and men.

In all race distances, HEATS showed stronger rank correlations (except for the first lap), indicating that rankings were more predictable in HEATS, whereas FINALS displayed greater fluctuations in positioning. This finding aligns with prior research that has observed similar differences between heats and finals. For example, Fang et al. (17) found that medallists adopted different pacing strategies in heats compared to finals, likely as an energy-conserving strategy to optimise performance in later rounds. Additionally, official competition rules strategically distribute the strongest swimmers across multiple heats primarily to maintain spectator interest and prevent favourites from competing against each other too early in the semifinals or finals. Heats are seeded based on entry times, with the slowest swimmers competing in the earliest heats and the fastest swimmers in the later heats of each round (1). This results in a more predictable ranking order within HEATS, as performance differences between swimmers are larger, leading to stronger rank correlations. It also shows that top swimmers establish their position fairly early thereafter maintaining control throughout the rest of the race. The strategy implies to secure an strong initial positioning rather than relying on a late explosive finish (16, 19). In contrast, FINALS bring together only the fastest qualifiers, creating a much more competitive environment where swimmers are more closely matched in ability. As a result, tactical decisions, pacing adjustments, and mid-race surges play a more prominent role, contributing to greater rank fluctuations (16). Furthermore, psychological factors such as increased pressure, race-day nerves, and heightened competition awareness may lead to more aggressive race strategies and positional changes throughout the event (12, 24, 25).

Across all race distances, rank correlations increased from the first to the final timing point, indicating greater stability in race rank order as the event progressed. Early segments of the race exhibited greater variability in ranking positions, particularly in middle-distance events: However, as swimmers settled into their pacing strategies, ranking became more consistent in the latter stages of the race. This finding aligns with prior research which shows that top athletes tend to position themselves optimally for the finish spurt (12, 13, 26). Further, other studies have shown that a more even pacing strategy is more effective than a positive pacing approach in certain endurance sports (12, 27, 28), indicating that top athletes regulate their effort more effectively by maintaining a steady pace and utilizing their superior fatigue resistance in the latter stage of the race. In contrast, lower-level athletes tend to start too aggressively in an attempt to match the pace of stronger competitors, which can result in premature fatigue and a subsequent decline in performance as the race progresses. This strategic approach likely explains the greater rank correlations observed toward the end of the race, as stronger swimmers execute their pacing strategies more effectively, securing their positions in the final stages. However, overconfidence or misjudging tactical positioning at key race segments can jeopardise medal chances. This is particularly relevant in long-distance events, where rank correlation coefficients reached 0.9 before the halfway mark. In other words, if swimmers are not in a competitive position by approximately 400 m in the 800 m or 1,500 m events, their chances of contending for a top finish may be significantly reduced.

The greater rank stability in 1,500 m at intermediate points, compared to the 400 m and 800 m, suggests that in endurance swimming, strategic positioning is established earlier. This could reflect a greater emphasis on even-paced or negative-split strategies, where swimmers deliberately conserve energy in the early segments to finish strongly (12, 28). Prior research has shown that swimmers in long-distance events tend to adopt a more controlled and economical pacing strategy, whereas middle-distance swimmers may engage in more mid-race tactical surges or variations in speed (12, 29). By the final lap, rank correlations converge across all distances, indicating that race order is largely established before the final lap, regardless of race length. This suggests that top swimmers secure their positions earlier in the race, emphasizing the importance of tactical positioning rather than late-race surges. These findings highlight the need for strategic pacing and well-timed positioning before the final segment, rather than relying solely on end-race efforts. Future research should examine how swimmers optimize their positioning strategies and the role of pacing, psychological readiness, and energy distribution in maintaining a competitive edge leading into the final lap.

Across all distances, the highest proportion of Top3 finishes occurred in the central lanes (4 and 5). Since these lanes are assigned to the fastest qualifiers, it is expected that swimmers in these lanes would have a higher likelihood of finishing in the Top3. However, the performance differences between lanes with similar seed times (e.g., lane 3 outperforming lane 6) suggest that factors beyond qualification speed influence race outcomes. One key explanation is wave turbulence and drafting effects (30, 31). Swimmers in central lanes may benefit from reduced wave drag, as they are surrounded by equally fast competitors on both sides (5, 6). Despite the greatest benefits are observed when drafting behind another swimmer, swimmers still benefit from lateral drafting (32, 33). When swimming at 1 m beside another swimmer and 0.5–1 m behind him (drafter head approximately at hip level of the leader), swimmers’ experience a 6%–7% drag reduction (32). This reduction translates into improved efficiency and could theoretically result in a 1.5%–3% performance gain (32). Conversely, water surface disturbances caused by waves from neighbouring swimmers may interfere with optimal streamlining and water catch at the beginning of the arm stroke (4). Compared to the outer lanes, however, the central lanes offer a better overview of competitors, which may further aid in optimal pacing and positioning within the race. The stronger performance of swimmers in lanes 6 and 7, compared to the outermost lanes (0 and 9 for HEAT; 1 and 8 for FIN), may also be linked to hydrodynamic advantages. While swimmers in the outermost lanes experience less wave turbulence from adjacent competitors, reflected waves from the pool walls may create additional inward-directed turbulence. Overall, these results highlight the existence of favourable lane assignments in competitive swimming. However, further research is needed to fully understand the effects of wave disturbance and drafting on performance.

Despite potential differences in absolute performance metrics (e.g., race times, stroke rates), the relative patterns of rank fluctuations, increasing stability over time, and the impact of lane placement on performance outcomes were relatively consistent between women and men. This aligns with previous research showing that, at the highest levels of competition, pacing strategies and race dynamics tend to be dictated more by event demands than by sex-specific physiological differences (34, 35). The lack of significant sex-based interactions indicates that both male and female swimmers experience similar competitive pressures and race evolution, particularly regarding the tactical shifts seen in finals compared to heats. Based on the current findings, coaches may apply similar principles for race execution for elite male and female swimmers.

## Practical applications

This study provides valuable insights for enhancing tactical preparation and race strategies for elite swimmers. The findings suggest that race dynamics differ significantly between heats and finals, with greater rank stability observed in heats and more fluctuations in finals. Coaches can leverage this information to tailor strategies for swimmers, emphasizing early positioning in heats to secure a strong start, while preparing for more flexible and adaptive pacing in the finals.

The complementary analyses also highlight the importance of lane placement. While this concept is well understood by coaches and athletes, offering data-backed statistical evidence and quantification of winning chances depending on the lane positioning provides additional practical value.

Furthermore, the analysis of rank progression and pacing strategies across different race distances emphasizes the importance of pacing consistency, particularly in longer events. Swimmers in these races should focus on maintaining a steady pace from the start, avoiding early surges that may lead to fatigue. Coaches can incorporate these pacing strategies into training, emphasizing the critical role of securing a strong position early, particularly in events like the 1,500 m, where strategic positioning early in the race is crucial for a top finish.

## Conclusion

This study highlights key differences in race dynamics, showing that HEATS exhibit greater rank stability than FIN, especially in events of 400 m and longer, likely due to reduced tactical variability. Rank stability increased as races progressed, suggesting that tactical positioning becomes more predictable as athletes settle into their pacing strategies. Long-distance events (800 m and 1,500 m) showed greater mid-race stability compared to shorter distances, however by the final stretch, rank order was similarly established across all events. Lane placement influenced Top3 finishes, with central lanes (4 and 5) offering the greatest advantage. Interestingly, lane 3 outperformed lane 6 despite similar seed times pointing to subtle lane-based performance factors. These trends were consistent across sexes, suggesting similar race dynamics for women and men. Our findings offer valuable insights into race strategy optimization, potentially helping athletes refine their tactical positioning and enhance performance outcomes. Future research should explore the physiological and psychological mechanisms underlying tactical positioning and pacing across different race types.

## Data Availability

Publicly available datasets were analyzed in this study. This data can be found here: https://www.swimrankings.net.
